# Predicting mortality in sick African children: the FEAST Paediatric Emergency Triage (PET) Score

**DOI:** 10.1186/s12916-015-0407-3

**Published:** 2015-07-31

**Authors:** Elizabeth C. George, A. Sarah Walker, Sarah Kiguli, Peter Olupot-Olupot, Robert O. Opoka, Charles Engoru, Samuel O. Akech, Richard Nyeko, George Mtove, Hugh Reyburn, James A. Berkley, Ayub Mpoya, Michael Levin, Jane Crawley, Diana M. Gibb, Kathryn Maitland, Abdel G. Babiker

**Affiliations:** Medical Research Council Clinical Trials Unit (MRC CTU) at UCL, London, UK; Department of Paediatrics, Mulago Hospital, Makerere University, Kampala, Uganda; Department of Paediatrics, Mbale Regional Referral Hospital, Mbale, Uganda; Department of Paediatrics, Soroti Regional Referral Hospital, Soroti, Uganda; Kilifi Clinical Trials Facility, KEMRI-Wellcome Trust Research Programme, Kilifi, Kenya; Department of Paediatrics, St Mary’s Hospital, Lacor, Uganda; Department of Paediatrics, Joint Malaria Programme, Teule Hospital, Muheza, Tanzania; Wellcome Trust Centre for Clinical Tropical Medicine and Department of Paediatrics, Faculty of Medicine, Imperial College, London, UK; Centre for Tropical Medicine and Global Health, Nuffield Department of Medicine, University of Oxford, Oxford, UK

**Keywords:** Africa, Children, FEAST trial, Mortality, Risk score

## Abstract

**Background:**

Mortality in paediatric emergency care units in Africa often occurs within the first 24 h of admission and remains high. Alongside effective triage systems, a practical clinical bedside risk score to identify those at greatest risk could contribute to reducing mortality.

**Methods:**

Data collected during the Fluid As Expansive Supportive Therapy (FEAST) trial, a multi-centre trial involving 3,170 severely ill African children, were analysed to identify clinical and laboratory prognostic factors for mortality. Multivariable Cox regression was used to build a model in this derivation dataset based on clinical parameters that could be quickly and easily assessed at the bedside. A score developed from the model coefficients was externally validated in two admissions datasets from Kilifi District Hospital, Kenya, and compared to published risk scores using Area Under the Receiver Operating Curve (AUROC) and Hosmer-Lemeshow tests. The Net Reclassification Index (NRI) was used to identify additional laboratory prognostic factors.

**Results:**

A risk score using 8 clinical variables (temperature, heart rate, capillary refill time, conscious level, severe pallor, respiratory distress, lung crepitations, and weak pulse volume) was developed. The score ranged from 0–10 and had an AUROC of 0.82 (95 % CI, 0.77–0.87) in the FEAST trial derivation set. In the independent validation datasets, the score had an AUROC of 0.77 (95 % CI, 0.72–0.82) amongst admissions to a paediatric high dependency ward and 0.86 (95 % CI, 0.82–0.89) amongst general paediatric admissions. This discriminative ability was similar to, or better than other risk scores in the validation datasets. NRI identified lactate, blood urea nitrogen, and pH to be important prognostic laboratory variables that could add information to the clinical score.

**Conclusions:**

Eight clinical prognostic factors that could be rapidly assessed by healthcare staff for triage were combined to create the FEAST Paediatric Emergency Triage (PET) score and externally validated. The score discriminated those at highest risk of fatal outcome at the point of hospital admission and compared well to other published risk scores. Further laboratory tests were also identified as prognostic factors which could be added if resources were available or as indices of severity for comparison between centres in future research studies.

**Electronic supplementary material:**

The online version of this article (doi:10.1186/s12916-015-0407-3) contains supplementary material, which is available to authorized users.

## Background

The admission burden to paediatric wards or the emergency room in African hospitals is very high, with many children presenting with complications of common infectious diseases such as sepsis and malaria. Life-threatening complications, including shock, are frequently present, and mortality is high, at 15–30 % [1–3]. Severe forms of pneumonia, sepsis, or malaria are amongst the most common causes of death [4], yet differentiation of the specific underlying cause is often unclear at point of admission since they share similar presenting clinical characteristics. Furthermore, most deaths occur within the first 24 h of admission [5]. The WHO integrated management guidelines recommend identifying children with ‘emergency’ or ‘priority’ features as a proxy for triage for immediate treatment in order to avert poor outcome; this approach has been shown to reduce mortality but implementation and consistency varies [6, 7]. At a clinical level, the key challenge facing health services in Africa is precisely how to distinguish those who are at greatest risk of poor outcome, using largely clinical criteria, in order to target parenteral antimicrobials and supportive therapies. Bedside clinical risk scores at admission can be used as part of triage systems to discriminate between children at high and low mortality risk. They can also be used to calculate risk-adjusted estimates of mortality in order to compare quality of care across hospitals or to stratify children entering clinical trials or other studies [8].

Paediatric risk scores have been developed in well-resourced countries, and are used to describe severity of illness in paediatric intensive care units in a variety of settings [9–12]. Examples include the Pediatric Risk of Mortality (PRISM) score, the Paediatric Index of Mortality score (PIM) [13, 14], the Pediatric Early Warning System score (PEWS), and Bedside PEWS [15]. These have helped with early identification for transfer to high dependency units and in research to enable between site and within study comparison of severity of illness. However, very few generic paediatric risk scores have been developed in resource-limited settings and those that are published have not been widely used or validated. The limited utility for general triage of some scores that focus on specific pathogens, such as malaria [16, 17] or meningococcal disease [18, 19], is due to the requirement of laboratory confirmation, therefore making them more suitable for retrospective risk stratification. Thus, there is a need for a practical risk score based only on clinical bedside measures that can be easily and quickly identified by busy healthcare workers on admission, that has an intuitive range, that does not need a specific disease or infection to be identified, that uses clinical signs that are found in populations in these settings, and that discriminates well between children at different risks of mortality. Such a score would also be useful in real-time stratification of children for trial enrolment and comparison of centres in these settings.

We identified prognostic factors for mortality in febrile children with signs of shock admitted to emergency care wards in East Africa and enrolled into the FEAST (Fluid Expansion as Supportive Therapy) trial, and used them to develop a bedside risk score for mortality. This score was then validated using data on children admitted to a rural district hospital in Kilifi, Kenya, and its performance compared to other published risk scores.

## Methods

### Study setting

Derivation data came from the FEAST trial which took place in six centres (both large regional referral hospitals and small district hospitals) across three countries (Kenya, Uganda, and Tanzania) from 2009 to 2011 and enrolled 3,170 sick febrile children aged between 2 months and 12 years with clinical evidence of impaired perfusion ([20], ISCRTN 69856593). FEAST was conducted in malaria endemic areas where national vaccination programmes included Haemophilus influenza type B vaccine, but not a pneumococcal vaccine. Prior and during the trial, admitting clinicians and nurses received Emergency Triage Assessment and Treatment training [21], which included the assessment of clinical features of shock. Eligible children had an abnormal temperature (pyrexia (≥37.5 °C) or hypothermia (<36 °C)), severe illness (presence of one or both of impaired consciousness (prostration, the inability of a child older than 8 months of age to sit upright or the inability of a child 8 months of age or younger to breast-feed; or coma, the inability to localize a painful stimulus) and respiratory distress) and clinical evidence of impaired perfusion (one or more of the following: capillary refill time >2 s; lower limb temperature gradient, defined as a notable temperature change from cold (dorsum of foot) to warm (knee) when running the back of hand from the toe to the knee; weak radial pulse or severe tachycardia, defined as heart rate >180 beats per min (bpm) for children <1 year old, >160 bpm for those 1 to 4 years old, >140 bpm for those ≥5 years old). Children with severe malnutrition, burns, trauma, gastroenteritis, or a presumed non-infectious cause of severe illness were excluded. Children were randomised to receive boluses of 20–40 mL/kg of 5 % human albumin solution or 0.9 % saline solution over one hour, or maintenance fluids only at 4 mL/kg/h (no bolus control group). Those with severe hypotension (systolic blood pressure <50 mmHg for those aged <1 year, <60 mmHg for those 1–4 years old, <70 mmHg for those ≥5 years old) were randomly assigned in a separate stratum to receive 40 mL/kg bolus of either albumin or saline. All children enrolled in both strata were included in this study. Standardised case report forms were completed at enrolment and at specific time points during the first 48 h. At enrolment, lactate, haemoglobin, oxygen saturation, and glucose were measured and an HIV antibody test and rapid diagnostic test for malaria were performed. An automated handheld blood analyser (i-STAT, Abbott Laboratories, Abbott Park, IL) was used for immediate analyses of pH level, potassium, base excess, blood urea nitrogen (BUN), sodium, chloride, TCO_2_, and PCO_2_. Children with haemoglobin <5 g/dL were routinely transfused according to national guidelines [22].

The validation data came from one of the FEAST trial sites, a rural district hospital in Kilifi, Kenya, which has a general paediatric ward and a high dependency ward. The Kenya Medical Research Institute Programme has established ward surveillance and used standardised forms to systematically collect clinical admission data on all infants and children entering the hospital wards since 1989, which has been linked to demographic surveillance in the district since 2002 [23]. Children were routinely transferred to the high dependency unit if they had impaired consciousness (prostration or coma) or deep-breathing (a clinical sign of metabolic acidosis), or if they required close medical supervision for life threatening complications such as status epilepticus, severe forms of shock, or a cardio-respiratory arrest. At admission to the high dependency unit (HDU) an extended set of clinical details were routinely collected.

The first validation datasets included children aged between 2 months and 12 years admitted to the general paediatric ward between March 2011 and December 2012 (5,173 children), and the second dataset is a subset of the first and includes all children contemporaneously admitted from the general ward to the HDU (1058/5173 children). These datasets did not include children from the FEAST trial, which finished enrolment at this centre in January 2011 and included information on the date, but not time, of death.

Other published paediatric risk scores were evaluated in the FEAST derivation and validation datasets. PRISM III was developed in paediatric intensive care units in the USA and has been validated in a variety of settings [10, 24–28]. The Bedside Pediatric Early Warning System score (PEWS) was developed in Canada to quantify severity of children in hospitalised children and help with referral to critical care experts [15]. For African paediatric populations, the AQUAMAT (African Quinine Artesunate Malaria Trial) prognostic score (0–5) was developed in a *post hoc* analysis from the trial dataset involving nine African countries as part of the AQUAMAT trial comparing anti-malarial treatments in children with severe malaria and included five parameters (base deficit, impaired consciousness, convulsions, elevated blood urea, and underlying chronic illness) which were independently associated with death [17, 29]. The Lamberéné Organ Dysfunction Score (LODS) was created using data from six African countries in children with malaria using only three parameters (deep breathing, coma, and prostration) [16, 30]. Berkley et al. [31] used Kilifi admission data from 1998 to 2001 to develop prognostic scores for deaths at different time points following admission, subsequently named during a published validation as the Pediatric Early Death Index for Africa (PEDIA). The AQUAMAT score has not been subject to external validation to date and PEDIA along with LODS have only recently been externally validated in Uganda in children with malaria and non-malarial illnesses [30].

### Statistical analyses

The prognostic model for mortality by 48 h was built following published guidelines [32] and is described in Additional file 1: Table S1. There were 315 deaths; thus, up to 30 candidate predictors could reasonably be considered [33]. Variables selected for initial consideration were measured in >95 % of the included children, had been found to be predictive in other studies, or were *a priori* thought to be clinically important, and not highly correlated with other variables (Table 1). All variables were measured at or within 1 h of randomisation, which occurred at a median (IQR) of 15 min (0–25 min) following ward admission, and prior to the administration of any trial intervention. Model derivation was based on multivariable fractional polynomials with backwards elimination using Cox proportional hazards regression in complete cases (adjusted for randomisation arm) [34]. Time to death was measured in hours and minutes (from the time of randomisation) and follow-up was censored at 48 h or time of leaving hospital if earlier. Cox proportional hazards regression was used to allow for information from children that absconded prior to 48 h to be included in the analysis (n = 11). This identified the most predictive variables for death and the best functional form for continuous variables (exit and non-linearity threshold *P* = 0.05). Interactions with the randomisation arm were also considered in this model. We carried out sensitivity analyses using logistic regression to build the model and restricting the derivation dataset to control arm data only (n = 1,044 children, 76 deaths).

**Table 1:** Candidate predictors of mortality considered for building multivariable model

Clinical bedside candidate predictors ^a^	Percentage missing	Laboratory candidate predictors ^a^	Percentage missing
Age (months)	0 %	Base excess (mmol/L)	34 %
Axillary Temperature (°C)	<1 %	Blood urea nitrate (mg/dL)	38 %
Capillary refill time (seconds)	<1 %	Chloride (mmol/L)	34 %
Conscious level ^b^	<1 %	Glucose (mmol/L)	6 %
Cough	<1 %	Haemoglobin (g/dL)	3 %
Lung crepitations ^c^	<1 %	HIV status	21 %
Decreased skin turgor	<1 %	Lactate (mmol/L)	5 %
Deep breathing	<1 %	Malaria positive	1 %
Fits greater than 30 min in this illness	1 %	Oxygen saturation (%)	4 %
Fits in this illness	<1 %	pCO_2_ (mmHg)	34 %
Fitting/convulsions at admission	1 %	pH	34 %
Heart rate (beats/min)	<1 %	Potassium (mmol/L)	35 %
History of fever	<1 %	Sodium (mmol/L)	33 %
Indrawing	<1 %	Systolic blood pressure	2 %
Jaundice	<1 %	TCO_2_ (mmol/L)	36 %
Liver size >2 cm below costal margin	<1 %		
Neck stiffness or bulging fontanelle	<1 %	Other predictors not considered	
Respiratory distress	<1 %	Mid-upper arm circumference	6 %
Respiratory rate (breaths/min)	<1 %		
Severe pallor ^d^	<1 %		
Sex	0 %		
Temperature gradient ^e^	0 %		
Vomiting	<1 %		
Weak pulse	0 %		
Weight (kg)	0 %		

^a^ Alphabetical order

^b^ Conscious level defined as prostrate (the inability of a child older than 8 months of age to sit upright or the inability of a child 8 months of age or younger to breast-feed) or coma (the inability to localize a painful stimulus)

^c^ Added breath sounds heard on inspiration in one or both lung fields: any one of crackles, clicks or rattling (rales)

^d^ Severe pallor manifested in tongue, gums, or inner eyelids

^e^ The temperature gradient was assessed by running the back of hand from the toe to the knee; a positive temperature gradient was defined as a notable temperature change from cold (dorsum of foot) to warm (knee)

A clinical bedside score (the FEAST Paediatric Emergency Triage (PET) score) was created by categorising the continuous variables using appropriate clinical cut-offs to use alongside already categorised variables in a Cox regression model. Coefficients for the categories of each variable in the model were then divided by the coefficient nearest zero and rounded to the nearest integer giving an initial score value [19]. These initial score values were then further modified to ensure a straightforward scale from 1–10 by assigning 2 to the initial value if it was >3, and 1 if it was ≤3, and dropping variables that added the least predictive ability to the model (assessed by using the Net Reclassification Index (NRI) [35]). A low score on this scale then indicated a low risk of mortality and a high score indicated a high risk of mortality.

The FEAST PET score was applied to the two validation datasets using the non-parametric area under the receiver operating curve (AUROC) to measure discriminative ability. Mortality was defined as death within 2 days of admission as time of death was not available in the two validation datasets. The FEAST data and two validation datasets were also used to validate other previously published scores. To validate the PEDIA score, immediate death (death within 4 h after admission, and calculated exactly in FEAST) was interpreted as death on the same day as admission, early death (death between 4 and 48 h) was interpreted as death within 2 calendar days of admission but not the same day, and late death (>48 h) as death occurring more than 2 days after admission. Calibration was measured by Hosmer-Lemeshow goodness-of-fit *χ*^2^ tests evaluated on groups defined by quintiles [36]. PRISM III, Bedside PEWS, AQUAMAT, and PEDIA scores were calculated using the available admission variables and unavailable variables in the scores were set to 0 (as recommended). Assessments at later time points were not available to use for PRISM III, although this score recommends using the worst clinical measurement in the first 24 h [13, 27].

We also considered whether laboratory candidate predictors (Table 1; with >5 % missing data) could improve the discriminatory ability of the score in situations where they could feasibly be measured (e.g. specific research studies). Multiple imputation by chained equations under the missing at random assumption, with predictive mean matching, was therefore used for imputation, including all factors in Table 1 in the imputation model and creating 25 imputed datasets [37]. Imputed and observed values were compared visually. The NRI [35] was calculated within each imputed dataset using mortality risk cut-offs at 5 %, 10 %, and 15 %, and the range and mean of this measure across the 25 imputed datasets was used to assess whether the additional laboratory variables could be usefully added to the clinical bedside variables already included in the score. The NRI assessed the ability of each additional variable to directly increase the discriminative ability of the model by looking at risk classification categories (with an increased NRI showing more children correctly classified). Backwards elimination (exit threshold mean *P* = 0.05 calculated from all imputed datasets) including all laboratory markers was then used to identify the laboratory variables with the largest NRIs across the imputed datasets. These were added to the clinical prognostic model to develop an extended score including laboratory markers identified as adding important information to risk scoring by the NRI. Rubin’s rules [38] were used to combine AUROCs from the multiply imputed datasets to validate the score including laboratory markers in the FEAST control arm data [39]. Finally, in an additional analysis, Cox regression was used to identify the best prognostic model for mortality based on best subsets regression in complete cases including all laboratory markers with <10 % missing data and considering all interactions. Statistical analyses were carried out in Stata (version 13.1).

## Results

Overall, 3,170 children with median age 24 months (IQR, 13–38) were recruited to the FEAST trial, of whom 315 (11 %) died within 48 h. A total of 3,121 (98 %) children (2,815 (99 %) surviving children and 306 (97 %) who died) had complete clinical data on admission for calculation of clinical bedside score. Of these, 15 % were comatose, 59 % had a temperature gradient, 51 % severe pallor (manifested in tongue, gums, or inner eyelids), and 21 % a weak pulse volume; median heart rate was 169 beats per minute (Table 2).

**Table 2:** Baseline characteristics of FEAST dataset and validation datasets from Kilifi

	FEAST (2009–2011)	Kilifi High Dependency Ward (2011–2012)	Kilifi General Admissions (2011–2012)
Baseline characteristics			
Number with FEAST PET score calculable (% dataset)	3125 (99 %)	1053 (99 %)	5098 (99 %)
Age (months), median (IQR)	24 (13–38)	38 (14–69)	24 (10–53)
Gender (Female, %)	1444 (46 %)	437 (42 %)	2149 (42 %)
Weight (kg) - median (IQR)	10 (9–13)	12 (8–16)	10 (7–14)
Conscious level – prostrate	1919 (61 %)	293 (28 %)	1117 (22 %)
– coma	463 (15 %)	311 (29 %)	331 (7 %)
Axillary temperature (°C), median (IQR)	38.2 (37.3–39)	37.6 (36.7–38.5)	37.6 (36.8–38.5)
History of fever (%)	3110 (99 %)	743 (71 %)	3560 (70 %)
Heart rate (beats per min), median (IQR)	169 (153–183)	140 (118–162)	144 (124–162)
Weak pulse (%)	660 (21 %)	77 (7 %)	98 (2 %)
Capillary refill time (s), median (IQR)	2 (1–3)	2 (1–2)	1 (1–2)
Temperature gradient (%)	1849 (59 %)	134 (13 %)	238 (5 %)
Respiratory rate (breaths per min), median (IQR)	58 (48–67)	40 (32–52)	38 (32–50)
Respiratory distress (%)	2585 (83 %)	288 (27 %)	1377 (27 %)
Deep breathing (%)	2019 (65 %)	211 (20 %)	369 (7 %)
Indrawing (%)	2129 (68 %)	229 (22 %)	1195 (23 %)
Lung crepitations (%)	692 (22 %)	139 (13 %)	633 (12 %)
Cough (%)	2245 (72 %)	328 (31 %)	2096 (41 %)
Severe pallor (%)	1588 (51 %)	412 (39 %)	1317 (26 %)
Convulsions (%)	455 (15 %)	359 (34 %)	1023 (20 %)
Decreased skin turgor (%)	187 (6 %)	89 (8 %)	338 (7 %)
Vomiting (%)	1603 (51 %)	348 (33 %)	1560 (31 %)
FEAST PET score, median (IQR) [range]	3 (2,4) [1, 9]	3 (1,4) [0,9]	2 (1,3) [0,9]

Twenty five variables were included in the model building process (Table 1) of which 10 were selected as independent predictors of mortality in the final model. The final prognostic model included axillary temperature, heart rate, weight, lung crepitations (added breath sounds heard on inspiration in one or both lung fields: any one of crackles, clicks or rattling (rales)), weak pulse, capillary refill time, conscious level, respiratory distress, deep breathing, and severe pallor. Identical independent predictors of mortality were also chosen using logistic regression (Additional file 1: Table S2). The strongest prognostic factors for mortality were coma, bradycardia (<80 beats per min), or severe tachycardia (>220 beats per min). Lower temperature and longer capillary refill times were also associated with an increased risk of death. Mortality risk increased as weight declined under <10 kg. As expected, weight and age were highly correlated (Spearman’s rho = 0.88, *P* <0.001), but age did not explain the mortality risk as well as weight (Akaike Information Criterion difference +4.8 for model including age rather than weight). Although weight-for-age z-score provided a similarly good model fit to weight, it was not included because it is not practical to calculate in an emergency setting. Weight and deep breathing in the presence of the other bedside factors were the least predictive (*P* >0.05 for their NRI values), and were therefore dropped in order to create a simple score ranging from 0–10 (Table 3). The FEAST PET score’s discriminative ability within the control arm (receiving maintenance fluids only, and selected as they were not affected by the adverse outcome of fluid boluses) of the FEAST derivation dataset was good with AUROC = 0.82 (95 % CI, 0.77–0.87) compared to 0.84 (95 % CI, 0.79–0.87) for full linear predictor from fitted regression coefficients, including all 10 variables and non-linearity. The median score in the control arm was 3 (IQR 2–4), while the maximum score was 9 out of a possible 10. Sensitivity analyses developing a score in the control arm data identified most of the 10 included variables as significant predictors, but failed to identify others with similar effect sizes in additional models due to reduced power (Additional file 1: Table S3).

**Table 3:** FEAST Paediatric Emergency Triage (PET) score and the FEAST Paediatric Emergency Triage and Laboratory (PETaL) score

Factor	Coefficient (95 % CI) from univariable model	Coefficient (95 % CI) from multivariable model ^a^	*P* value from multivariable model	Score value given if present	Maximum possible value for PET score	Maximum possible value for PETaL score
Axillary temperature: ≤37 °C	1.08 (0.86–1.31)	0.63 (0.38–0.87)	<0.001	1	1	1
Heart rate: <80 bpm (bradycardia)	2.46 (2.00–2.93)	1.34 (0.92–1.77)	<0.001	2	2	2
≥80 to <105 bpm	1.12 (0.57–1.68)	0.70 (0.11–1.30)	0.02	1		
≥220 bpm (severe tachycardia)	1.44 (0.73–2.14)	1.34 (0.92–1.77)	<0.001	2		
Capillary refill time: 2 or more seconds	0.93 (0.63–1.23)	0.53 (0.21–0.85)	0.001	1	1	1
Conscious level: prostrate	1.01 (0.58–1.46)	0.68 (0.23–1.13)	0.003	1	2	2
– coma	2.24 (1.80–2.69)	1.53 (1.06–2.00)	<0.001	2		
Respiratory distress	0.93 (0.52–1.34)	0.55 (0.07–1.02)	0.02	1	1	1
Lung crepitations	0.77 (0.55–1.01)	0.60 (0.36–0.85)	<0.001	1	1	1
Severe pallor	0.90 (0.66–1.14)	0.49 (0.22–0.76)	<0.001	1	1	1
Weak pulse	1.45 (1.24–1.68)	0.73 (0.48–0.97)	<0.001	1	1	1
Weight: <6 kg	0.52 (0.02–1.03)	0.41 (–0.05–0.88)	0.08	–	–	
6–8 kg	0.30 (–0.01–0.61)	0.21 (–0.03–0.45)	0.09			
Deep breathing	1.17 (0.86–1.49)	0.42 (0.06–0.77)	0.02	–	–	
Total					10	
Additional laboratory values to be added if measured						
Lactate >5 mmol/L	1.82 (1.54–2.09)	1.12 (0.79–1.46)	<0.001	2		2
pH <7.2	1.80 (1.51–2.08)	0.97 (0.69–1.25)	<0.001	1		1
Blood urea nitrogen >20 mg/dL	1.23 (0.95–1.52)	0.58 (0.26–0.90)	<0.001	1		1
Total (laboratory score)						14

^a^ Coefficient from linear predictor of multivariable cox regression model on complete cases. First section includes clinical factors only. Second section (laboratory values) adjusted for all clinical factors

Note: weight and deep breathing were the least predictive factors and were therefore excluded from the score. Univariable models and multivariable model also adjusted for randomisation arm which was not included in the score

Multiple imputation with chained equations was used to assess the potential for the laboratory candidate predictors in Table 1 to add important information to a risk score, even if these might be evaluated on a smaller number of children. The NRI calculated in 25 imputed datasets with risk category cut-offs of 5 %, 10 %, and 15 % identified lactate, HIV status, TCO_2_, potassium, pH, BUN, and base excess as variables that significantly improved the mortality score (Additional file 1: Table S2). Using backwards elimination including the clinical factors and all laboratory markers, lactate, BUN, and pH added independent information to the score (lactate NRI range 10.7–14.2 %, mean *P* <0.001, BUN NRI range 2.8–8.9 %, mean *P* = 0.02, pH NRI range 4.8–9.1 %, mean *P* = 0.03; Additional file 1: Table S4). The NRI of adding all three laboratory variables to the clinical score was 24.7–28.9 %, all *P* <0.001. These variables were therefore categorised using appropriate clinical cut-offs and added to the FEAST score extending the range of the score to 0–14 (Table 3) and creating the FEAST Paediatric Emergency Triage and Laboratory (PETaL) score. The AUROC for the FEAST PETaL score in the control arm from the multiply imputed data was 0.86 (95 % CI, 0.82–0.90). Oxygen saturation, although considered important in other studies, was not shown to significantly improve the discriminative ability of the score in our dataset (NRI range 1.1–5.3 %, mean *P* = 0.08; Additional file 1: Table S4).

**Fig. 1:**
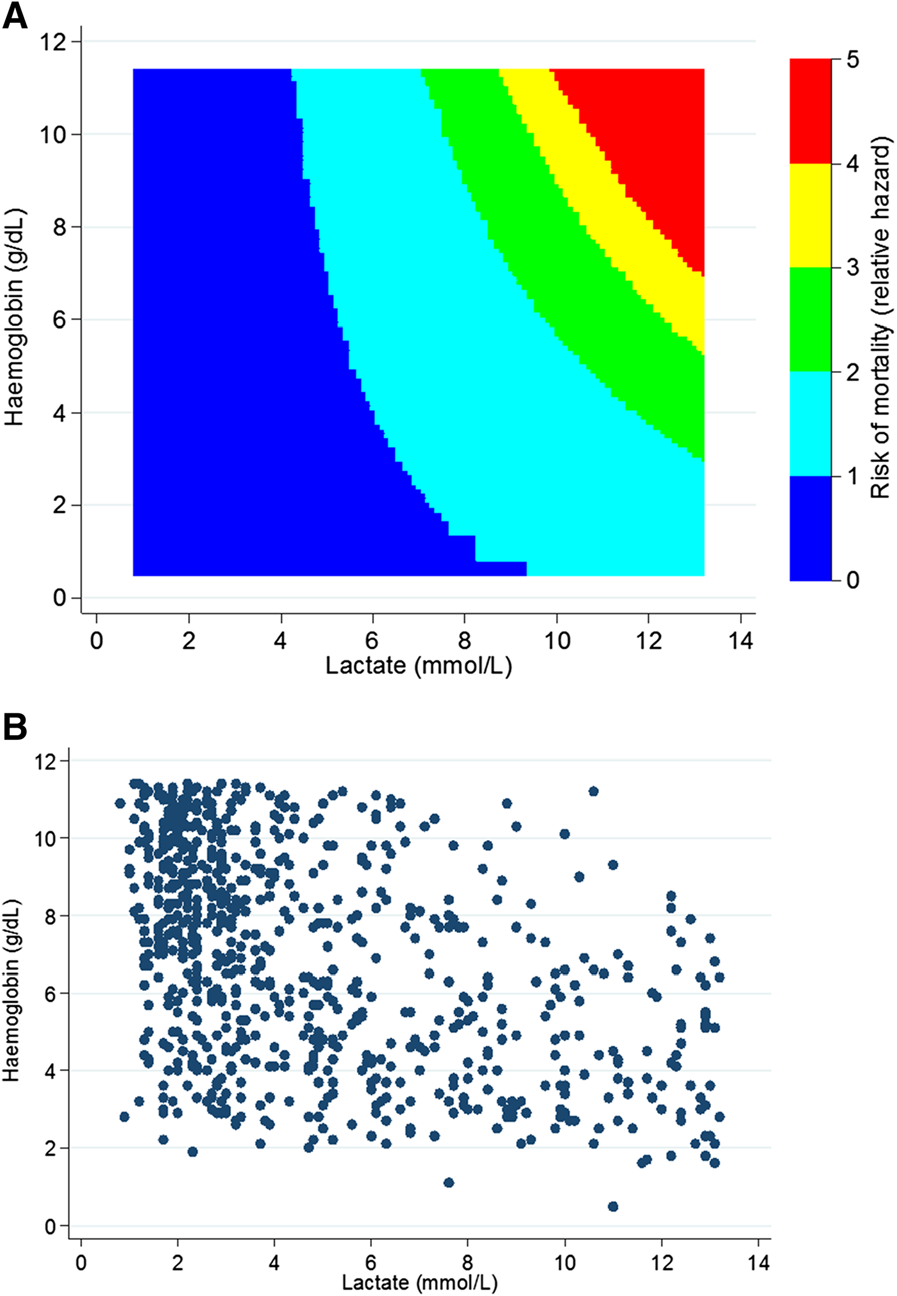
Plots of the relationship between haemoglobin and lactate and mortality estimated from the adjusted Cox regression model. **a** Contour plot of mortality risk by baseline haemoglobin and lactate estimated from Cox regression model. This shows in contrast to risk uniformly increasing with increasing lactate and uniformly decreasing with haemoglobin as might have been expected, we have observed increased risk (green to red) when haemoglobin is increasing and lactate is increasing relative to an average child enrolled who had a lactate of 5 mmol/L and a haemoglobin of 7 g/dL. **b** Scatter plot of observed baseline haemoglobin and lactate values

A Cox regression analysis using best subsets regression and including the 10 clinical variables from the model and the laboratory candidate predictors with <10 % missing data on complete cases only, identified lactate, haemoglobin, glucose, and malaria test results to be important additional predictors of mortality (although notably these factors did not all increase the ability to distinguish mortality risk between children). A positive malaria test result and high glucose were associated with a reduced mortality risk (Additional file 1: Table S5). We found an interaction between haemoglobin and lactate: rather than the mortality risk uniformly increasing with increasing lactate and uniformly decreasing with increasing haemoglobin, the higher risk associated with higher lactate (>7 mmol/L) values was restricted to those with high haemoglobin (>6 g/dL). For children with profound anaemia (haemoglobin <4 g/dL) there was a similar risk regardless of lactate level, compared to an average child enrolled who had a lactate of 5 mmol/L and a haemoglobin of 7 g/dL (Fig. 1).

**Fig. 2:**
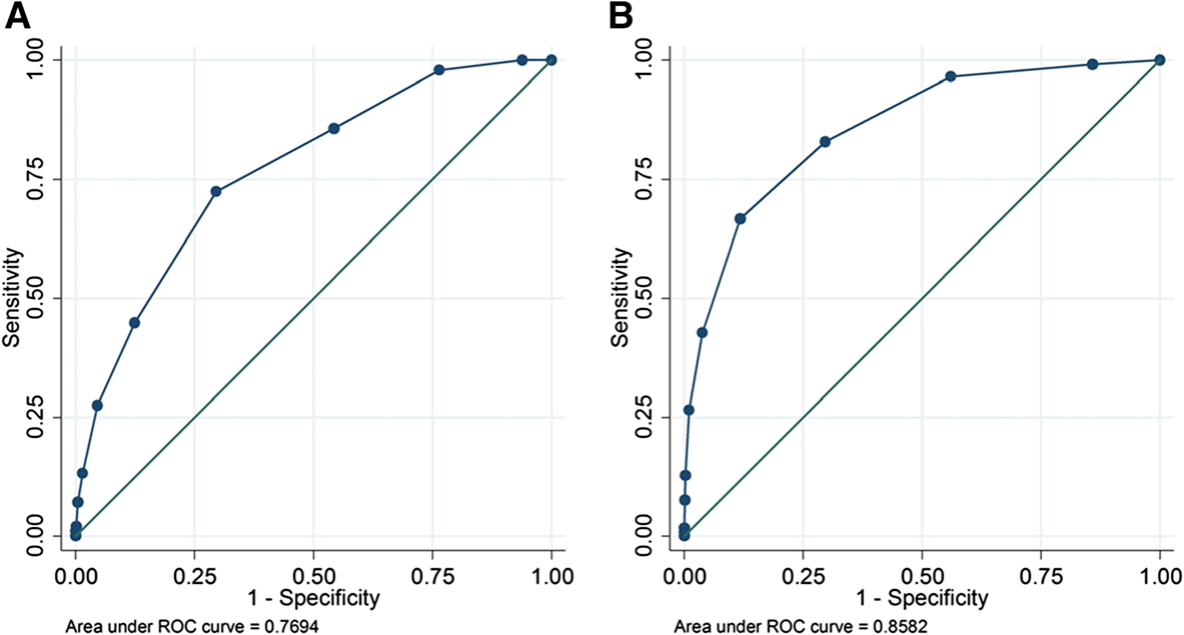
Receiver operating characteristic curves for the FEAST PET score in (**a**) the Kilifi high dependency unit and (**b**) the Kilifi general admissions dataset

The FEAST PET score was externally validated on the Kilifi HDU data (1,053 children, 98 (9 %) deaths), and showed a fair discriminative ability with AUROC of 0.77 (95 % CI, 0.72–0.82) and Hosmer-Lemeshow test *P* = 0.30 indicating good fit. The score’s discriminative ability improved in the general admissions dataset (5,098 children, 117 (2 %) deaths) giving an AUROC of 0.86 (95 % CI, 0.82–0.89) and Hosmer-Lemeshow test *P* = 0.51 (Fig. 2).

**Fig. 3:**
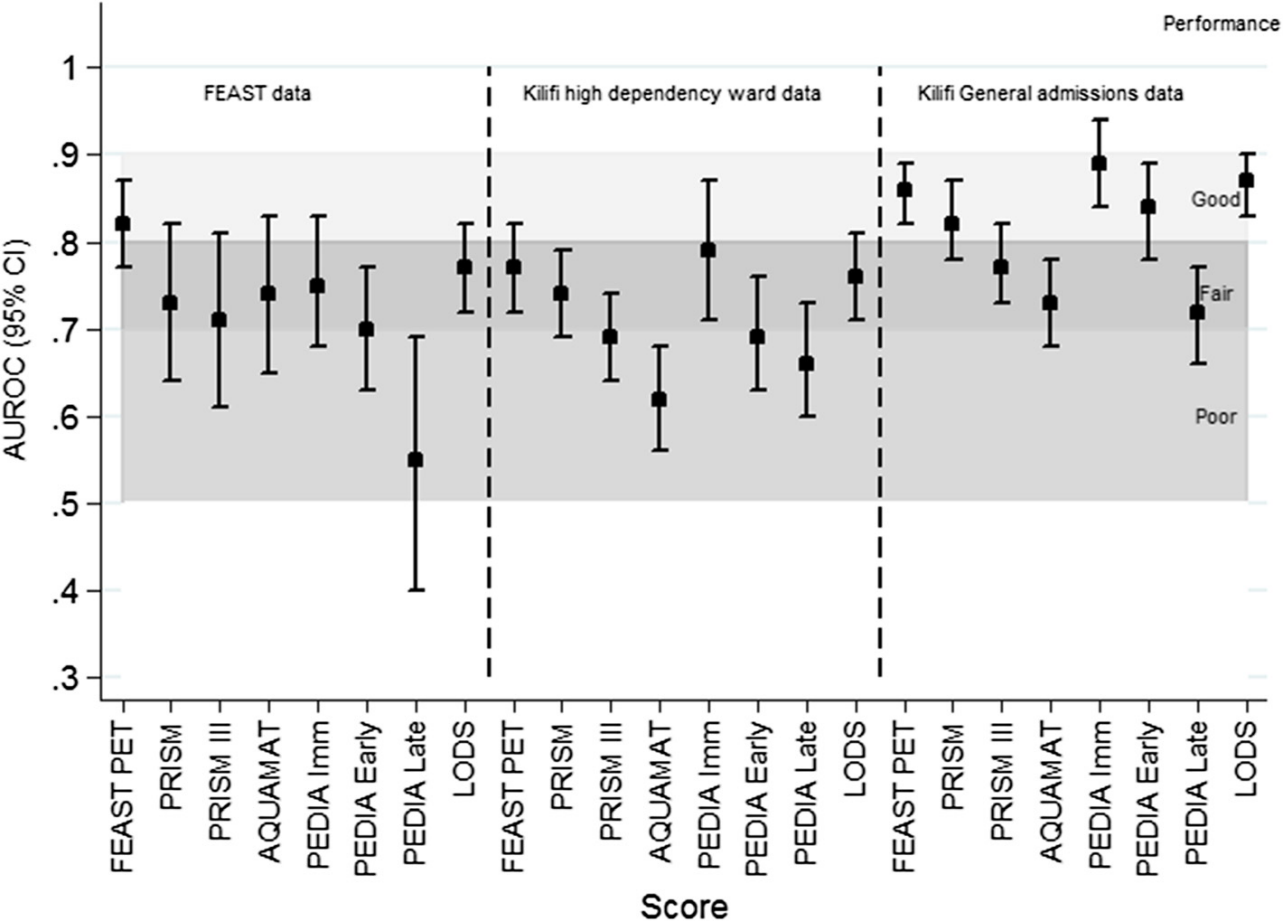
Discriminatory ability of different scores when applied to data from FEAST and Kilifi

In comparison with other scores, the FEAST PET score showed significantly better discriminative ability than Bedside PEWS, PRISM III, and the AQUAMAT scores (Fig. 3; *P* <0.05 test for equality between AUROC scores), and no evidence for a difference for LODS, and PEDIA on the two validation datasets (Table 4). LODS discriminated well in all the validation datasets and gave an AUROC of 0.76 (0.71–0.81) in the HDU and 0.87 (0.83–0.90) in the general admissions dataset. The AQUAMAT trial score discriminated better when restricted to children with malaria in the FEAST trial (AUROC 0.80; 95 % CI, 0.68–0.93) but did not discriminate as well when restricted to the Kilifi datasets.

**Table 4:** Discriminatory ability of different scores when applied to data from FEAST and Kilifi

		FEAST (data from the control (no fluid bolus) arm only) (n = 1044)			Kilifi High Dependency Ward (n = 1058)			Kilifi General Admissions (n = 5107)		
Score	Variables included	Number with score (% died)	AUROC (95 % CI) ^e^	Hosmer-Lemeshow test (*P* value)	Number with score (% died)	AUROC (95 % CI) ^e^	Hosmer-Lemeshow test (*P* value)	Number with score (% died)	AUROC (95 % CI) ^e^	Hosmer-Lemeshow test (*P* value)
FEAST PET score	Axillary temperature, heart rate, capillary refill time, conscious level, deep breathing, respiratory distress, lung crepitations, weak pulse	1024 (7 %)	0.82 (0.77–0.87)	0.56	1053 (9 %)	0.77 (0.72–0.82)	0.30	5098 (2 %)	0.86 (0.82–0.89)	0.50
Bedside PEWS score ^a^	Heart rate, capillary refill time, respiratory rate, oxygen saturation, systolic blood pressure	1000 (9 %)	0.64 (0.56–0.71)	0.46	1053 (9 %)	0.69 (0.64–0.75)	0.56	5094 (2 %)	0.74 (0.69–0.79)	0.22
PRISM III score ^b^	Heart rate, temperature, conscious level, systolic blood pressure, glucose, potassium, PCO_2_ , pH, acidosis, pupillary reflexes	627 (6 %)	0.71 (0.61–0.81)	0.26	1056 (9 %)	0.69 (0.64–0.74)	0.10	5099 (2 %)	0.77 (0.73–0.82)	0.01
AQUAMAT score ^c^ (overall)	Conscious level, chronic disease, convulsions, BUN, and base excess	648 (5 %)	0.74 (0.65–0.83)	0.84	1011 (9 %)	0.62 (0.56–0.68)	0.79	4964 (2 %)	0.73 (0.68–0.78)	0.04
AQUAMAT score ^c^ (malaria positive only)	Conscious level, chronic disease, convulsions, BUN, and base excess	360 (3 %)	0.80 (0.68–0.93)	0.65	355 (6 %)	0.54 (0.42–0.66)	0.83	781 (3 %)	0.60 (0.49–0.72)	0.41
PEDIA Immediate death score ^d^	Anaemia, jaundice, indrawing, deep breathing, conscious level, convulsions/seizures, temperature	1007 (3 %)	0.75 (0.68–0.83)	0.64	680 (4 %)	0.79 (0.71–0.87)	0.47	3504 (1 %)	0.89 (0.84–0.94)	0.15
PEDIA Early death score ^d^	Jaundice, indrawing, conscious level, convulsions/seizures, wasting, kwashiorkor	1003 (4 %)	0.70 (0.63–0.77)	0.02	1016 (9 %)	0.69 (0.63–0.76)	0.76	5071 (2 %)	0.84 (0.78–0.89)	0.08
PEDIA Late death score ^d^	History >7 days, conscious level, convulsions/seizures, temperature, wasting, kwashiorkor	959 (1 %)	0.55 (0.40–0.69)	0.35	664 (10 %)	0.66 (0.60–0.73)	0.34	3472 (2 %)	0.72 (0.66–0.77)	0.08
LODS	Deep breathing, coma and prostration	1038	0.77 (0.72–0.82)	0.38	1057 (9 %)	0.76 (0.71–0.81)	0.62	5103	0.87 (0.83–0.90)	0.74

^a^ Variables in the score but not measured: receipt of oxygen therapy, respiratory effort in four categories (normal, mild increase, moderate increase, severe increase, any apnoea). Underlined variables were available in the FEAST dataset but not in the Kilifi datasets

^b^ Variables in the score but not measured: pupillary reflexes, pH, total CO_2_, PCO_2_, arterial PaO_3,_ creatinine, urea, white blood cells, prothrombin time, and platelets. Underlined variables were available in the FEAST dataset but not in the Kilifi datasets

^c^ Underlined variables were available in the FEAST dataset but not in the Kilifi datasets

^d^ Time of death was not available in the Kilifi data. Immediate deaths were defined as those that occurred on the same day as admission to hospital. The early death score was calculated on mortality by two calendar days but not the same day as admission. Late death defined as strictly greater than 2 days after admission. Immediate deaths were not included in the early death analysis, immediate and early deaths were not included in the late death analysis as in the original publication

^e^ The AUROC value for each score was compared to the FEAST PET score for mortality by 48 h. In the FEAST dataset there was no evidence of a difference between the AUROC for the FEAST PET score versus the AQUAMAT score overall (*P* = 0.19) and in malaria only (*P* = 0.65), and the FEAST PET score was significantly better than Bedside PEWS (*P* < 0.001), PRISM III (*P* = 0.02), LODS (*P* = 0.05), and PEDIA for immediate (*P* = 0.002) and early death (*P* = 0.04). In the Kilifi validation datasets (high dependency/general) there was no evidence of a difference between the AUROC for the FEAST PET score versus LODS (*P* = 0.67/0.73) or PEDIA for immediate (*P* = 0.34/0.82) and early (*P* = 0.63/0.47) death, and the FEAST PET score was significantly better than Bedside PEWS (*P* = 0.02/<0.001), PRISM III (*P* = 0.003/<0.001), and the AQUMAT scores (*P* <0.001/<0.001)

AQUAMAT, African Quinine Artesunate Malaria Trial; AUROC, Area under the receiver operating curve; BUN, Blood urea nitrogen; FEAST, Fluid Expansion as Supportive Therapy; LODS, Lamberéné Organ Dysfunction Score; PEDIA, Pediatric Early Death Index for Africa; PET, Paediatric Emergency Triage; PEWS, Pediatric Early Warning System; PIM, Paediatric Index of Mortality; PRISM, Pediatric Risk of Mortality

## Discussion

Herein, we have developed and externally validated a bedside clinical risk score for severely ill children presenting to emergency care wards in resource-limited settings in Africa that identifies those at greatest risk of mortality within 48 h of admission. The FEAST PET score is straightforward to use, includes only clinical variables that are measured at the bedside, does not rely on laboratory tests, and is not limited to children with specific diagnoses, but rather covers different presentation syndromes reflecting the population of children presenting to hospital in these settings.

Prognostic scores created in resource-limited settings have not often been externally validated, even though this is an important part of the development process [40]. This may explain why previous scores have not been widely implemented. The FEAST PET score had fair discriminative ability for HDU data and good discriminative ability for general admissions data, showing that it is generalizable to other clinical settings. The two validation cohorts were heterogeneous, one included more critically sick children that had been transferred to a HDU which also acted as a research ward and the other a more general paediatric admission population of which the majority had come through the emergency room. We have already highlighted the clinical and epidemiological challenge of differentiating the major causes of childhood illnesses since many have overlapping clinical presentations [41–43]. Interestingly, the FEAST PET score discriminated best in the general admissions dataset, likely because this included more children with low scores with very low mortality risk (Additional file 1: Figure S1). However, similar proportions with high scores died in both general admissions and HDU validation sets, demonstrating that the FEAST PET score is able to identify those children at particularly high mortality risk even within children presenting to the emergency room with a diverse set of underlying conditions. However, perhaps because of the diversity of underlying conditions among children in the FEAST trial, the FEAST PET score discriminated best in the general admissions dataset. This suggests that the emergency room or general admissions would be the most appropriate setting in which to explore implementation, perhaps in comparison to the simpler LODS score.

Comparing different scores for 48 h mortality in the two validation datasets, the FEAST PET score performed similarly well to the PEDIA immediate death and early death score, and the LODS score, and better than Bedside PEWS, PRISM III, and AQUAMAT scores, likely due to the FEAST PET score’s good generalisability and because all of the variables were easily recorded at the bedside. It is perhaps not surprising that the PEDIA immediate and early death scores performed well within these datasets, as PEDIA was based on data previously obtained from the same hospital, but it is interesting that the very simple LODS score (based on only three factors) also performed well. The three PEDIA scores have only been externally validated once in Uganda [30] and may be complex to implement since different prognostic factors predict scores for different times of death (immediate vs early vs late) and have different weighting within each score. The PEDIA score for late deaths (>48 h) in particular discriminated poorly.

A limitation of our validation is that many of the laboratory tests included in PIM and PRISM III, and commonly done in well-resourced settings (such as total bilirubin, calcium, potassium, arterial oxygen tension, creatinine, prothrombin time) are not measured in most African countries, and were not available in the FEAST or validation datasets. The PIM score could not be validated at all as none of its variables were recorded in the validation dataset [14]. The AQUAMAT score performed well in the FEAST trial subgroup with malaria, but performed poorly in the Kilifi validation dataset probably because two of the five severity measures in the score were not routinely recorded (BUN and base excess).

Nevertheless, it is interesting to consider how much additional discrimination could be obtained from wider use of laboratory test results in resource limited-settings. In our further analyses we added three laboratory measures (lactate, BUN, pH) to the PET score based on their NRI to create the PETaL score; however, this did not change discriminatory power in the derivation dataset, showing that clinical measures can be sufficient for a good score, and that improving prediction does not always improve ability to discriminate children at low and high risk. Unfortunately, we were not able to externally validate the PETaL score as the laboratory measures were not available in the Kilifi validation datasets.

However, the fuller prognostic model including laboratory tests (with <10 % missing data) can provide insights into underlying epidemiology of the acutely sick child in Africa. For example, we found that the increased risk of death associated with higher lactate, i.e. >7 mmol/L, was much greater among children with haemoglobin >6 g/dL. All those with low haemoglobin (<4 g/dL) had similar risk regardless of lactate, possibly because 89 % of these severely anaemic children were effectively treated with blood transfusion [22], whereas those with high haemoglobin had diverse underlying causes. This may also be due to different causes of high lactate in anaemic (reduced oxygen carrying capacity) compared to non-anaemic patients manifesting cardiovascular compromise of septic shock with diminished oxygen delivery (leading to anaerobic metabolism from shock) [44]. Moreover, acute onset of severe anaemia (to levels as low as 5 g/dL) can be well tolerated because of compensatory mechanisms to sustain tissue oxygenation [45].

Similarly, the association between reduced mortality and a positive malaria test in the present study may be due to asymptomatic *P. falciparum* infection being common in malaria endemic areas, and carrying lower overall mortality, especially compared to children with bacterial infections [46, 47]. Of interest, oxygen saturation, a predictor of mortality in other studies [48, 49], was not a significant predictor in our dataset, which may be due to its effect being captured by other clinical measures. Furthermore, the limited predicative ability of hypoxia compared to another clinical feature (crepitations) for identifying children with probable pneumonia supports WHO recommendations of the value of this sign reinforcing the diagnosis of pneumonia in children with severe breathing difficulties. Although crepitations could be considered a subjective sign dependent on the observer, a sensitivity analysis showed that excluding it worsened the discriminatory ability of the score (AUROC without lung crepitations 0.80 (0.75–0.86); *P* = 0.04 in FEAST control arm data). This indicates it is important to retain in the bedside score.

Advantages of using the FEAST dataset to develop a clinical bedside score are its large size, multi-centred and multi-disease nature with substantial subgroups with severe malaria and sepsis, and its high quality as it was collected during a randomised controlled trial [50, 51] with few missing data for bedside measures. The pragmatic nature of the trial design enabled it to be carried out in centres without a history of research and without many interventions at a site level. The standard-of-care for trial participants was thus very similar to the standard-of-care on the wards, but with increased monitoring by nurses over the first 48 h of admission. One important limitation is that, despite it being a useful prognostic factor in other studies [52], we were not able to consider mid-upper arm circumference in any analysis due to differences in data completeness between survivors and non-survivors (violating the missing at random assumption needed for multiple imputation), probably due to mid-upper arm circumference being of low priority to complete immediately upon admission.

We developed the FEAST PET score using the full clinical trial dataset (315 deaths) including intervention arm as a model factor in order to increase power. However, this raises potential concerns about interactions with randomised interventions: the alternative strategy is to restrict derivation models to the control arm only (76 deaths) with consequent power reductions. As no significant or important interactions had been identified [20], we chose the former. Repeating the score derivation process on control arm data as a sensitivity analysis, as suggested by a reviewer, gave broadly similar results, but un-intuitively identified fits as significantly protective and, despite similar effect sizes, failed to identify capillary refill time and pallor as significant predictors (Additional file 1: Table S3).

## Conclusions

Although it would benefit from external validation in a multi-centre African population outside of the FEAST trial centres before implementation, there are several ways that the FEAST PET score could be used. One would be as an inclusion criterion for clinical trials, or to stratify children into groups or perform risk-adjusted comparisons of emergency care. For research studies recording laboratory data, the FEAST PETaL score could be used for inter-site or inter-centre comparisons. However, potentially the most valuable use of this standardised, validated score is to support the implementation of triage in resource-limited routine care settings, thereby facilitating rapid prioritisation of care, or closer monitoring, for the sickest children and hence improved outcomes. Improved triage has been shown to reduce mortality in these settings [6] and the FEAST PET score would work across specific syndromes and specific diseases to identify those that need prioritisation of any supportive therapies available. It would also help ensure consistent comparisons between patients by clinicians, compared to simple clinical opinion, and encourage better examination of clinical signs by all staff. Having a score that is simple to implement and uses commonly measured clinical signs could increase the number of hospitals in resource-limited settings that successfully implement the triage process.

## Additional file

Additional file 1:
**Supplementary Tables and Figures.**

## Abbreviations

AQUAMAT: African Quinine Artesunate Malaria Trial
AUROC: Area under the receiver operating curve
BUN: Blood urea nitrogen
FEAST: Fluid Expansion as Supportive Therapy
HDU: High dependency unit
LODS: Lamberéné Organ Dysfunction Score
NRI: Net Reclassification Index
PEDIA: Pediatric Early Death Index for Africa
PET: Paediatric Emergency Triage
PETaL: Paediatric Emergency Triage and Laboratory
PEWS: Pediatric Early Warning System score
PIM: Paediatric Index of Mortality score
PRISM: Pediatric Risk of Mortality
