# Supplementary material for: Predicting mortality in sick African children: the FEAST Paediatric Emergency Triage (PET) Score
Source: BMC Med. 2015 Jul 31;13:174. doi: 10.1186/s12916-015-0407-3 (PMC4521500; doi:10.1186/s12916-015-0407-3)
Supplement: Additional file 1: — Supplementary Tables and Figures. [file 12916_2015_407_MOESM1_ESM.docx]

Additional File 1

Table 1: Prognostic model building decisions.

| Issue | Decision |
| --- | --- |
| Selecting appropriate predictors | Selected those found in other studies, reliably measured, and considered a-priori by clinicians to be important. |
| How to include continuous predictors | Used fractional polynomials. This was done with the multivariable fractional polynomial command in Stata with sequential selection algorithm and alpha set at 0.05. |
| How to deal with missing data | Used multiple imputation (MI) for laboratory variables where missing data were >5%. After examining missingness of additional variables the missing at random assumption was considered to hold and MI could be used to create 25 imputed datasets. |
| Choosing model building method | Used Cox regression as times of death were available. Censored at the first of death, absconding from hospital or 48 hours from randomisation. |
| Selection method and criterion for final model | Used stepwise backwards elimination with a p-value of 0.05 as the selection criteria. |
| Assessing interactions in the final model | Interactions with the randomisation arm were assessed amongst pre-specified predictors. No strong interactions were found (p<0.01). |
| Assessing performance of model | AUROC and Hosmer-Lemeshow test in FEAST dataset control arm data only |
| Validating model | AUROC and Hosmer-Lemeshow test in Kilifi high dependency ward and general admissions datasets |

Table 2. Comparing score derived from logistic regression to that derived from Cox Proportional hazards regression.

|  | Coefficient (95% CI) from multivariable model | |
| --- | --- | --- |
| Variable | Cox proportional hazards | Logistic regression |
| **Axillary temperature: ≤37°C** | 0.63 (0.38-0.87) | 0.69 (0.40-0.99) |
| **Heart rate:**  **<80bpm (bradycardia)** | 1.34 (0.92-1.77) | 2.05 (1.13-2.98) |
| **≥80- <105bpm** | 0.70 (0.11-1.30) | 1.02 (0.21-1.82) |
| **≥220bpm (severe tachycardia)** | 1.38 (0.92-1.77) | 1.63 (0.53-2.74) |
| **Capillary refill time (CRT): 2 or more seconds** | 0.53 (0.21-0.85) | 0.52 (0.16-0.88) |
| **Conscious level: prostrate** | 0.68 (0.23-1.13) | 0.81 (0.30-1.31) |
| **coma** | 1.53 (1.06-2.00) | 1.80 (1.26-2.33) |
| **Respiratory distress** | 0.55 (0.07-1.02) | 0.75 (0.21-1.30) |
| **Lung crepitations** | 0.60 (0.36-0.85) | 0.80 (0.50-1.11) |
| **Severe pallor** | 0.49 (0.22-0.76) | 0.55 (0.24-0.87) |
| **Weak pulse** | 0.73 (0.48-0.97) | 0.77 (0.47-1.06) |
| **Weight: <6kg**  **6-8kg** | 0.41 (-0.05-0.88)  0.21 (-0.03-0.45) | 0.40 (-0.24-1.0)  0.07 (-0.3-0.45) |
| **Deep breathing** | 0.42 (0.06-0.77) | 0.46 (0.06-0.86) |

|  | Main model (clinical bedside score) | | Model selected in control arm | | Union of main and control arm model variables | |
| --- | --- | --- | --- | --- | --- | --- |
|  | Full dataset | Control arm dataset | Full dataset | Control arm dataset | Full dataset | Control arm dataset |
| **Ax. temperature: ≤37°C** | 0.63  (0.38, 0.87) | 0.72  (0.23, 1.22) | 0.63  (0.39, 0.86) | 0.70  (0.21, 1.18) | 0.61  (0.36, 0.85) | 0.70  (0.19, 1.20) |
| **Heart rate:**  **<80bpm (bradycardia)**  **≥80- <105bpm**  **≥220 bpm (severe tachycardia)** | 1.34  (0.92, 1.77)  0.70  (0.11, 1.30)  1.34  (0.92, 1.77) | 1.63  (0.76, 2.50)  0.19  (-1.27, 1.64)  1.63  (0.76, 2.50) | 1.38  (0.62, 1.10)  0.53  (-0.06, 1.12)  1.38  (0.62, 1.10) | 2.23  (1.45, 3.01)  -0.24  (-1.68, 1.19)  2.23  (1.145, 3.01) | 1.34  (0.91, 1.76)  0.72  (0.13, 1.31)  1.34  (0.91, 1.76) | 2.06  (1.20, 2.92)  -0.03  (-1.50, 1.43)  2.06  (1.20, 2.92) |
| **CRT : 2 or more seconds** | 0.53  (0.21, 0.85) | 0.60  (-0.11, 1.31) | - |  | 0.49  (0.17, 0.82) | 0.53  (-0.18, 1.23) |
| **Conscious level: prostrate**  **coma** | 0.68  (0.23, 1.13)  1.53  (1.06, 2.00) | 0.53  (-0.38, 1.44)  1.71  (0.78, 2.65) | 0.79  (0.34, 1.23)  1.76 (1.27,2.26) | 0.78  (-0.11, 1.67)  2.23  (1.45, 3.01) | 0.71  (0.25, 1.16)  1.64  (1.14, 2.13) | 0.69  (-0.22, 1.60)  2.32  (1.33, 3.30) |
| **Respiratory distress** | 0.55  (0.07, 1.02) | 0.17  (-0.77, 1.10) | - |  | 0.53  (0.06, 1.01) | 0.14  (-0.82, 1.09) |
| **Lung crepitations** | 0.60  (0.36, 0.85) | 0.79  (0.27, 1.10) | 0.61  (0.37, 0.85) | 0.80  (0.30, 1.31) | 0.60  (0.35, 0.84) | 0.84  (0.31, 1.37) |
| **Severe pallor** | 0.49  (0.22, 0.76) | 0.55  (-0.03, 1.13) | - |  | 0.45  (0.18, 0.73) | 0.43  (-0.16, 1.01) |
| **Weak pulse** | 0.73  (0.48, 0.97) | 0.80  (0.29, 1.31) | 0.86 (0.62,1.10) | 0.84  (0.35, 1.34) | 0.73  (0.48, 0.98) | 0.70  (0.19, 1.21) |
| **Weight:**  **<6kg**  **6-8kg** | 0.41  (-0.05, 0.88)  0.21  (-0.03, 0.45) | 0.34  (-0.62, 1.29)  0.13  (-0.38, 0.64) | - |  | 0.40  (-0.07, 0.87)  0.21  (-0.04, 0.45) | 0.56  (-0.37, 1.47)  0.24  (-0.27, 0.76) |
| **Deep breathing** | 0.42  (0.06, 0.77) | 0.62  (-0.18, 1.42) | 0.74  (0.42, 1.07) | 0.94  (0.26, 1.63) | 0.42  (0.06, 0.76) | 0.64  (-0.18, 1.46) |
| **Fits at admission** |  |  | -0.47  (-0.80,-0.14) | -1.39  (-2.14, -0.64) | -0.24  (-0.58, 0.10) | -1.31  (-2.11, -0.51) |

Table 3: Sensivity analysis building a model using FEAST control arm data only rather than the whole clinical trial dataset as the derivation data.

Figure 1: A) Distribution of FEAST PET, LODS and PEDIA immediate scores and B) estimated mortality at each score value in Kilifi general admissions data and Kilifi High Dependency ward data.


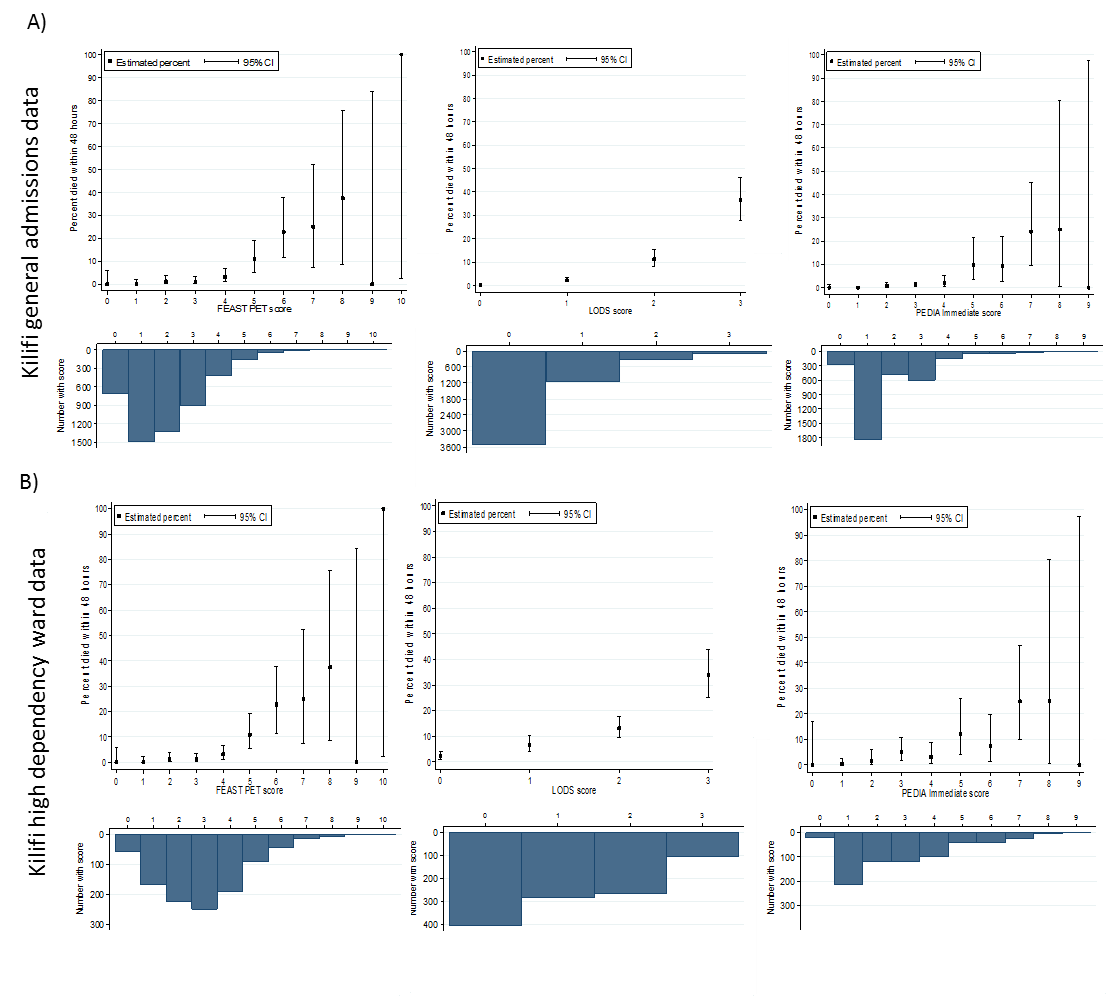


Table 4: Net Reclassification Index ranges across 25 imputed datasets for candidate laboratory markers when added individually and in combination to the clinical model.

| Univariable analyses (added individually to clinical model) |  |  |  |
| --- | --- | --- | --- |
|  | NRI range | Two-sided p-value range | Mean p-value |
| Lactate | 20.4-23.1% | <0.001 | <0.001 |
| TCO_2_ (mmol/L) | 18.2-23.0% | <0.001 | <0.001 |
| pH | 13.8-19.7% | <0.001 | <0.001 |
| BUN | 9.9-16.4% | <0.001 | <0.001 |
| Base excess | 18.3-23.4% | <0.001 | <0.001 |
| Potassium | 6.3-11.8% | <0.001-0.03 | 0.003 |
| HIV positive | 2.4-6.0% | 0.004-0.2 | 0.03 |
| Glucose | 2.8-5.3% | 0.015-0.2 | 0.08 |
| Oxygen Saturation | 1.1-5.3% | 0.001-0.4 | 0.08 |
| Malaria test positive** | 2.3-5.4% | 0.02-0.3 | 0.1 |
| Systolic Blood Pressure | 2.0-3.4% | 0.02-0.2 | 0.1 |
| Haemoglobin | 1.1-3.3% | 0.04-0.6 | 0.2 |
| Chloride | 1.2-7.6% | 0.002-0.6 | 0.2 |
| PCO_2_ | -0.9-4% | 0.01-1.0 | 0.3 |
| Sodium | -1.1-2.3% | 0.07-1.0 | 0.6 |
| Factors identified through backwards elimination process, included multivariably*. |  |  |  |
| Lactate | 10.6-16.7% | <0.001 | <0.001 |
| BUN | 3.1-8.2% | <0.001-0.11 | 0.02 |
| pH | 2.9-9.0% | <0.001-0.22 | 0.03 |
|  |  |  |  |
| Combined effect of Lactate, BUN and pH | 24.6-28.9% | <0.001 | <0.001 |

* NRI’s calculated from one multivariable model considering each factor separately, and then adding all three

together to the clinical model to estimate the NRI’s for a combined effect.

***Plasmodium falciparum* malaria slide or rapid diagnostic test positive

Table 5: Cox regression coefficients of prognostic model including laboratory variables found using best subsets regression.

| Variable | Coefficient (95% CI) | p-value |
| --- | --- | --- |
| **Axillary temperature (°C)** | -0.12 (-0.22, -0.01) | p=0.03 |
| **Heart rate^2 (bpm)** | -1.61 (-2.3, -0.95) | p<0.001 |
| **Heart rate^2*log(heartrate) (bpm)** | 1.49 (0.79, 2.20) | p<0.001 |
| **Capillary refill time (s)** | 0.03 (-0.12, 0.18) | p=0.71 |
| **Conscious level: prostrate** | 0.37 (-0.10, 0.85) | p=0.13 |
| **coma** | 1.14 (0.62, 1.66) | p<0.001 |
| **Respiratory distress** | 0.60 (0.13, 1.07) | 0.01 |
| **Crackles** | 0.57 (0.30, 0.84) | p<0.001 |
| **Severe pallor** | 0.35 (-0.07, 0.76) | p=0.10 |
| **Weak pulse** | 0.53 (0.24-0.81) | p<0.001 |
| **Weight (kg)** | -0.01 (-0.05, 0.21) | p=0.47 |
| **Deep breathing** | 0.09 (-0.28, 0.47) | p=0.62 |
| **Lactate (mmol/l)** | 0.06 (-0.003, 0.12) | p=0.07 |
| **Haemoglobin (g/dl)** | -0.05 (-0.15, 0.06) | p=0.35 |
| **Lactate and haemoglobin interaction term** | 0.02 (0.007, 0.02) | p<0.001 |
| **log(glucose) (mmol/l)** | -0.38 (-0.56, -0.20) | p<0.001 |
| **Malaria test positive** | -0.76 (-1.01, -0.50) | p<0.001 |
